# SABDR: Bidirectional Dynamic Domain Adaptation with Style Alignment for Small Object Detection Under Adverse Weather

**DOI:** 10.3390/s26123626

**Published:** 2026-06-06

**Authors:** Wei Tang, Xuekai Zhang, Yueping Peng, Hexiang Hao, Zecong Ye, Le Li, Yingying Sun

**Affiliations:** 1 School of Information Engineering, Engineering University of PAP, Xi’an 710086, China; 13414667546@163.com (W.T.); 18510004596@163.com (X.Z.); hhx1214s@163.com (H.H.); x.cat18509783@163.com (L.L.); 19591579025@163.com (Y.S.); 2 Unit Command Department, Officers College of PAP, Chengdu 610213, China; yzc6666@yeah.net

**Keywords:** adverse weather, small object detection, unsupervised domain adaptation, style alignment, cross-domain object detection

## Abstract

Small object detection under adverse weather remains challenging due to weather-induced domain shifts and sparse visual cues of small targets. In contrast to R-YOLO/QTNet and conventional UDA methods, which mainly rely on weather-specific restoration/enhancement or global feature/magnitude alignment, SABDR explicitly targets cross-weather small object adaptation through bidirectional domain translation, degradation-aware receptive-field modeling, feature-statistics modulation, and style-direction alignment. Specifically, the Bidirectional Dynamic Domain Adaptation Network, termed BiDDC-Net, translates between source and target domains and dynamically adjusts receptive fields according to weather severity. The Style-Aware Domain Adaptation Module, termed AIFI-DA, enhances discriminative small-object channels using feature statistics. SDA is further used as a complementary training-time regularizer to encourage style-direction consistency without directly matching feature magnitudes. Experiments are conducted on Cityscapes→Foggy Cityscapes and MOT-Fly→Foggy/Rainy/Snowy MOT-Fly, including newly added rainy and snowy MOT-Fly settings, with both YOLOv5s and YOLO26 evaluated on all MOT-Fly weather conditions. SABDR achieves 47.7 ${\mathrm{mAP}}_{50}$ on Cityscapes→Foggy Cityscapes, and obtains 96.0%/96.8%, 66.7%/77.1%, and 95.0%/95.6% ${\mathrm{mAP}}_{50}$ on Foggy, Rainy, and Snowy MOT-Fly with YOLOv5s/YOLO26, respectively. The improvements on MOT-Fly are reported under a fixed single-seed setting and should therefore be interpreted as single-run empirical gains rather than statistically validated improvements. These results demonstrate its effectiveness under the evaluated fog/rain/snow cross-weather small object detection settings.

## 1. Introduction

In emerging visual perception tasks such as low-altitude security patrols, drone emergency rescue, and low-altitude traffic control, the detection of small targets under adverse weather conditions has become a critical bottleneck [1]. Taking urban low-altitude security as an example, it is necessary to accurately identify small unauthorized drones under foggy conditions, and this work further evaluates rain and snow degradation in the newly added MOT-Fly rain/snow settings, to prevent such targets from posing a threat to critical facilities such as airports [2,3]. Such scenarios face cross-domain shifts from “normal weather” to “adverse weather,” instantiated in this study by the evaluated fog setting and rain/snow settings; due to the extremely small target scale and sparse feature dimensions [4], they impose stricter requirements on the cross-domain adaptability of detection algorithms compared to more general targets.

In recent years, deep learning-based object detection technologies have made significant progress in single-domain scenarios under normal weather conditions [5]. However, when models are directly applied to the evaluated adverse-weather settings, including MOT-Fly fog, rain and snow settings, significant distribution shifts exist between the source domain (normal weather) and the target domain (adverse weather) due to degraded image quality, e.g., reduced contrast, blurred textures [6], rain streaks, or snow-induced occlusions, leading to a sharp decline in detection performance. This issue can easily trigger accidents in safety-critical low-altitude perception tasks, highlighting the urgency of developing robust cross-domain detection technologies.

This issue is particularly pronounced for small targets such as drones [7]. On the one hand, small targets have a low pixel proportion and sparse features, requiring sophisticated feature extraction for accurate identification even under normal weather conditions [8]; on the other hand, the evaluated adverse-weather settings further blur their edges and textures while amplifying background interference, such as light spots in foggy conditions and rain streaks or snow-induced occlusions [9]. This results in the “sparsity of small-target features” and “cross-domain distribution shifts” compounding to create a dual detection challenge. In cross-domain scenarios, small targets are typically more prone to being missed than medium- or large-sized targets; this phenomenon is particularly pronounced in low-altitude drone scenarios [10], and thus significantly impacts the reliability of low-altitude perception systems.

Existing cross-domain target detection methods are primarily designed for general-sized targets and have inherent limitations in small-target scenarios [11]. Unsupervised domain adaptation methods based on adversarial alignment reduce domain differences through global feature alignment; however, global alignment tends to drown out the sparse features of small targets in background information, leading to the loss of semantic information for small targets [12]. Methods based on image translation can generate cross-domain images [13,14], but their style transfer process is optimized independently of the detection task, failing to ensure the preservation of key features such as edges and structures of small targets [15]. Transformer-based methods, while possessing global modeling capabilities, rely on self-attention mechanisms that compute correlations on a global scale [16], which tends to dilute the local features of small targets. These issues indicate that cross-domain detection methods designed for general-purpose targets are difficult to directly transfer to scenarios “small target + adverse weather” [17], necessitating the development of specialized cross-domain adaptation mechanisms tailored to the characteristics of small targets. These issues indicate that cross-domain detection methods designed for general-purpose targets are difficult to directly transfer to “small-target + evaluated adverse-weather” scenarios, including the fog, rain and snow settings considered in this work, necessitating specialized cross-domain adaptation mechanisms tailored to the characteristics of small targets. The object-size statistics of MOT-Fly further confirm that the UAV targets occupy only a small portion of the image area, which makes this benchmark suitable for evaluating small-object detection under weather-induced domain shifts.

**Figure 1:**
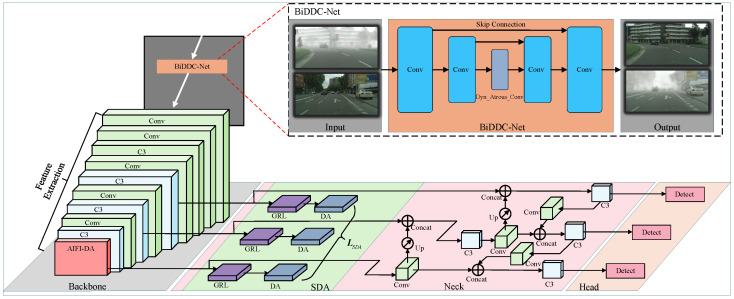
Overall Architecture of the SABDR Algorithm. The figure illustrates the core workflow: input image pairs undergo preprocessing via BiDDC-Net (image processing module), followed by multi-scale feature extraction via the Backbone (which incorporates domain-adaptation branches). The Neck then fuses features into a feature pyramid, with the Head ultimately outputting object detection results. This diagram also highlights the collaborative logic among key modules such as domain adaptation (DA) and feature interaction (Concat/Up).

To address these challenges, this paper proposes SABDR (Bidirectional Dynamic Domain Adaptation and Style Alignment for Small Object Detection under Adverse Weather Conditions), a cross-domain detection method tailored for small object detection in adverse weather. The main contribution of SABDR is a coordinated design of input-side sample augmentation, feature-side style modulation, and loss-side directional constraints for adverse-weather small-object domain adaptation. Different from R-YOLO/QTNet and existing UDA detectors, SABDR extends the QTNet-style single-direction translation to BiDDC-Net with bidirectional source-target translation, replaces fixed/ordinary convolution with degradation-aware dynamic dilated convolution, and introduces style direction alignment instead of conventional feature or magnitude alignment. Through the joint optimization of data augmentation, feature adaptation, and style alignment, SABDR addresses feature sparsity, domain shift, and style mismatch in cross-domain small-object detection under adverse weather. Its overall framework is shown in Figure 1. The main contributions of this paper are as follows.

1. Bidirectional Dynamic Domain Adaptation Network (BiDDC-Net): To address the challenges posed by significant variations in image degradation under adverse weather conditions—where a fixed receptive field struggles to balance contextual modeling with the preservation of small-object details—this paper proposes the Bidirectional Dynamic Domain Adaptation Network (BiDDC-Net). During training, this module expands cross-domain samples through bidirectional domain transformation. In the generation branch, it introduces a degradation-aware dynamic dilated convolution mechanism that adaptively adjusts the size of the receptive field based on changes in the input image’s brightness and contrast. When degradation is severe, the model incorporates richer contextual information; when degradation is mild, it prioritizes preserving the local structure and textural details of small objects. In this way, BiDDC-Net provides more stable cross-domain inputs for subsequent feature adaptation.
2. Style-Aware Domain Adaptation Module (AIFI-DA): To address style shifts in mid-to-high-level features caused by adverse weather conditions, as well as the tendency for discriminative information such as small object edges and textures to be weakened during global alignment, this paper proposes the lightweight style-aware domain adaptation module AIFI-DA. Embedded between the backbone network output and the neck, this module extracts style vectors from feature statistics to dynamically modulate conditional normalization parameters and spatial encodings, while utilizing style-guided feature interaction to enhance discriminative channels related to small objects. This design helps preserve the semantic consistency and local structural information of small objects while adapting to the target domain’s style.
3. Style Direction Alignment Loss (SDA): To address the issue that traditional alignment losses directly constrain the numerical proximity of source-domain and target-domain features—which may impose excessive restrictions on the sparse representation of small objects—this paper introduces SDA as a complementary training-time regularizer (SDA). This loss constructs style direction vectors from multi-scale features and uses cosine similarity to constrain the consistency of style variation trends between the source and target domains, without unduly restricting their amplitude differences. SDA shares the style vector representation with AIFI-DA, forming a synergistic mechanism of “feature modulation + loss constraint”.

To validate the effectiveness of the proposed method, experiments were conducted on two types of “clear weather → dense fog” cross-domain scenarios. First, Cityscapes [18] → Foggy Cityscapes [19] serves as a public standard benchmark to verify the method’s general adaptability across weather domains. Second, MOT-Fly [20] → Weather MOT-Fly, including Foggy, Rainy, and Snowy MOT-Fly, was used as a small-object UAV benchmark to evaluate the robustness of SABDR under different adverse-weather conditions. Experimental results demonstrate that SABDR achieves consistent improvements across these settings. On Cityscapes → Foggy Cityscapes, SABDR achieves an ${\mathrm{mAP}}_{50}$ of 47.7, representing a 2.5 percentage point improvement over R-YOLOv5. On Foggy, Rainy, and Snowy MOT-Fly, SABDR obtains ${\mathrm{mAP}}_{50}$ scores of 96.0/96.8, 66.7/77.1, and 95.0/95.6 with YOLOv5s/YOLO26 (https://docs.ultralytics.com/zh/models/yolo26) (accessed on 3 June 2026), respectively. These results indicate that the proposed method has good adaptability for cross-weather small-object detection under the evaluated fog/rain/snow settings.

## 2. Related Work

### 2.1. Overview of General Object Detection

Object detection methods have evolved from early traditional approaches that relied on manually designed features to detection frameworks primarily based on deep learning. Current mainstream methods mainly include two-stage detectors and one-stage detectors [21,22]. Two-stage methods, represented by Faster R-CNN, offer high detection accuracy; one-stage methods, represented by the YOLO series and RetinaNet [23,24], strike a good balance between detection speed and accuracy, and are therefore widely used in real-time visual perception tasks.

Building on this foundation, small object detection has gradually become an important branch of object detection. Due to their low pixel proportion and weak feature representation, small objects rely more heavily on high-resolution shallow-layer features and multi-scale fusion capabilities. Existing research typically enhances small-object representation through methods such as feature pyramids, detail enhancement, and attention modeling. However, most of these methods are designed for scenarios with high intra-domain clarity. When faced with adverse weather conditions and cross-domain distribution shifts, the edge, texture, and position information of small objects is more prone to degradation, leading to a significant decline in detection performance. Therefore, cross-domain detection of small objects under adverse weather conditions still requires further research.

### 2.2. Unsupervised Domain Adaptation Methods

Unsupervised Domain Adaptation (UDA) for object detection typically assumes that the source domain is labeled, while the target domain lacks human-provided labels [25]. Its objective is to improve the detector’s performance on the target domain without utilizing target domain labels. In recent years, UDA has emerged as a key research direction for enhancing the cross-domain generalization capabilities of object detection.

Existing UDA object detection methods can be broadly categorized into three types. The first category consists of feature alignment methods, which reduce the differences between the source and target domains by aligning distributions at the image-level [26], instance-level, or multi-scale feature-level. Representative methods are typically based on adversarial learning or statistical matching and have achieved good results in general object detection tasks. The second category comprises image translation methods, which transform source-domain images into the style of the target domain to narrow the visual differences between the two domains, thereby enhancing the detector’s adaptability to the target domain [27]. The third category consists of self-training or teacher-student methods, which gradually optimize the detector using target-domain pseudo-labels to enhance the model’s discriminative power in the target domain [28].

Although the aforementioned methods have made significant progress in general cross-domain detection tasks, most are designed for targets of conventional sizes and remain inadequate for scenarios involving small targets. Specifically, feature alignment methods are prone to background dominance, causing the weak features of small targets to be overwhelmed during global alignment; image translation methods, if lacking constraints specific to the detection task, tend to distort the edges and fine-grained structures of small objects; self-training methods rely on the quality of pseudo-labels, but small objects are more prone to missed detections and low confidence scores under adverse weather conditions, thereby limiting their adaptability. Therefore, cross-domain adaptation for small-object detection under adverse weather conditions still requires more targeted designs.

### 2.3. Cross-Domain Detection of Small Objects Under Adverse Weather Conditions

Cross-domain transfer from normal to adverse weather conditions has been a significant research direction in object detection in recent years, particularly holding high practical value for autonomous driving and low-altitude perception tasks. Compared to general objects, small objects—due to their low pixel count, weak semantic information, and strong reliance on local details—are more prone to missed detections and false positives under degraded conditions such as fog, rain, and snow. Consequently, the combination of adverse weather and small object detection further complicates the cross-domain challenge.

Existing research primarily improves detection performance under adverse weather conditions through image enhancement, domain alignment, and cross-domain feature modeling. For example, IA-YOLO [29] improves detection performance in foggy and low-light scenes via adaptive enhancement; R-YOLO [30] enhances the cross-domain capabilities of single-stage detectors [31] through pixel-level and multi-scale adversarial alignment; and methods such as DATR [32] attempt to enhance cross-domain feature modeling capabilities using Transformers. Furthermore, datasets such as M3D and methods like NSN provide data and noise suppression support for small-object cross-domain detection. It should be noted that, due to differences in task settings, detector architectures, and evaluation protocols, these methods mainly serve as related references in this paper, while the direct fair comparison is primarily conducted among Source-only, R-YOLO, and the proposed SABDR under the same experimental setting.

However, most of these methods are designed for general-purpose objects or overall scenes, and they do not sufficiently account for the feature fragility of small objects under adverse weather conditions. Global enhancement or global alignment tends to degrade fine-grained information such as the edges and textures of small objects; fixed receptive fields struggle to adapt to varying degrees of weather degradation; and global attention-based modeling, although effective for long-range dependency learning, may not always sufficiently preserve weak small-object cues when background interference is strong. Therefore, cross-domain detection of small objects under adverse weather conditions still requires more targeted designs. R-YOLO improves adverse-weather detection through pixel-level enhancement and adversarial alignment, but it does not explicitly target the sparse and fragile features of small objects. Transformer-based UDA methods, such as DATR, enhance cross-domain feature modeling with global attention, while image-translation-based methods reduce appearance gaps through style transfer. However, these methods may differ in their focus and may not simultaneously address weather degradation, small-object feature sparsity, and style mismatch. It should be noted that these comparisons are mainly methodological rather than strictly equivalent experimental comparisons, since different methods may use different backbones, detector architectures, training protocols, and computational budgets. Therefore, published results are treated as reference comparisons, while the fair comparisons in this paper are conducted among Source-YOLO, R-YOLO, and SABDR under the same setting.

In contrast, SABDR is designed for adverse-weather small-object UDA by combining BiDDC-Net, AIFI-DA, and SDA to jointly handle input degradation, feature style shifts, and fragile target discrimination. The goal is to provide a targeted collaborative adaptation mechanism for this challenging scenario.

## 3. Method

### 3.1. Problem Definition and Overall Framework

This paper investigates the problem of cross-domain detection of small targets under adverse weather conditions. By Equations (1) and (2), the source-domain and target-domain datasets are defined, respectively, as

(1) $$D_{s}={\left\{\left(x_{i}^{s},y_{i}^{s}\right)\right\}}_{i=1}^{N_{s}},$$

(2) $$D_{t}={\left\{x_{j}^{t}\right\}}_{j=1}^{N_{t}}.$$

Here, $x_{i}^{s}$ denotes the source-domain image, $y_{i}^{s}$ denotes the corresponding detection annotation, and $x_{j}^{t}$ denotes the target-domain image, for which no ground-truth annotations are provided. In this paper, the source domain corresponds to normal weather conditions, whereas the target domain corresponds to adverse weather conditions such as fog, rain, and snow. The objective is to learn a detector that achieves better detection performance in the target domain using only source-domain annotations and unlabeled target-domain images, with a particular emphasis on improving the detection of small objects under adverse weather conditions. It should be noted that this paper follows the standard unsupervised domain adaptation framework, aiming to alleviate cross-domain adaptation difficulties when target-domain annotations are scarce, rather than completely eliminating the costs of data collection and annotation in new scenarios.

To this end, we propose the SABDR framework. Building upon baseline cross-domain detectors, SABDR introduces two main modules and one complementary regularizer, namely BiDDC-Net, AIFI-DA, and SDA, to model small-object cross-domain detection under adverse weather conditions at the input/sample level, feature level, and training-objective level, respectively. The overall procedure is as follows. First, BiDDC-Net is used to enhance the adaptability of cross-domain samples and to adjust the receptive field according to the degree of image degradation. Subsequently, AIFI-DA is introduced during feature extraction to perform style-aware modulation on mid-to-high-level features. Finally, SDA is employed during training to constrain the consistency of multi-scale style directions between the source and target domains, thereby alleviating the over-constraint that may result from relying solely on direct numerical alignment.

Specifically, BiDDC-Net is primarily used to mitigate appearance differences and variations in degradation intensity caused by adverse weather. This module expands cross-domain training samples through bidirectional domain translation and combines this process with degradation-aware dynamic dilated convolutions, enabling the network to adaptively adjust its receptive field under different weather degradation conditions, thereby balancing the preservation of local details and the modeling of contextual information. AIFI-DA is mainly used to address style drift in mid-to-high-level features. This module extracts style vectors from feature statistics and uses them to modulate the normalization and feature interaction processes, thereby mitigating style mismatch caused by adverse weather while preserving discriminative features related to small objects as much as possible. SDA, as an additional constraint introduced during training, constructs style-direction vectors from multi-scale features and promotes alignment between the source and target domains in terms of style variation trends through directional consistency constraints, thereby providing a more stable optimization objective for feature modulation.

Therefore, SABDR does not rely solely on a single module to achieve cross-domain adaptation. Instead, it leverages the coordinated effects of degradation-aware enhancement on the input side, style modulation on the feature side, and directional constraints on the loss side, aiming to strike a better balance between cross-domain adaptation and the preservation of small-object features. Section 3.2, Section 3.3 and Section 3.4 below introduce the detailed designs of BiDDC-Net, AIFI-DA, and SDA, respectively.

### 3.2. BiDDC-Net (Bidirectional Dynamic Domain Adaptation Network)

BiDDC-Net is an improved version of the QTNet branch in the baseline R-YOLO and is designed for input-side cross-domain sample augmentation during the training stage. It focuses on appearance/style transformation between weather domains, such as brightness, contrast, texture, and fog-related visual characteristics, while preserving the original geometric structure, object layout, and bounding-box annotations. Unlike QTNet, which only performs unidirectional domain translation, BiDDC-Net simultaneously learns the mappings from the source domain to the target domain and from the target domain to the source domain. By Equation (3), the bidirectional mappings are formulated as

(3) $${\hat{x}}^{s\to t}=G_{s\to t}\left(x^{s}\right),\;{\hat{x}}^{t\to s}=G_{t\to s}\left(x^{t}\right).$$

**Figure 2:**
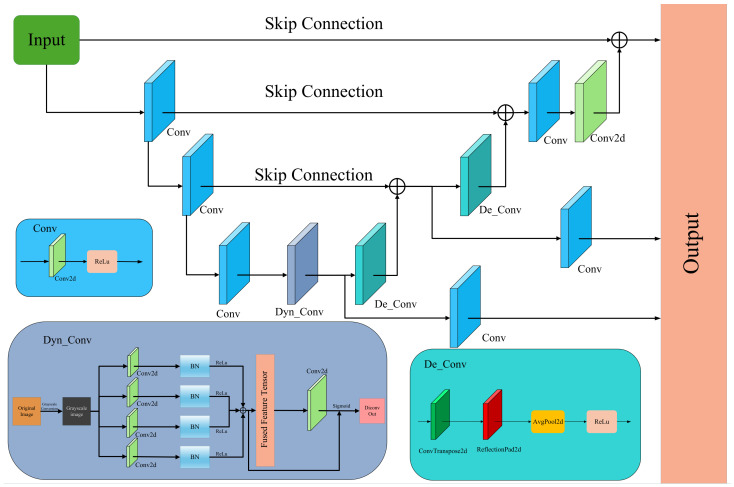
Schematic diagram of the BiDDC-Net architecture. The network enables bidirectional domain transformation of input images via multi-level convolutions and skip connections. Its core module, the Dyn-Conv, dynamically adjusts atrous convolution parameters based on image degradation level to enhance feature adaptation across weather conditions. Outputs include final results, facilitating multi-stage domain adaptation and cross-domain data generation.

Here, $x^{s}$ and $x^{t}$ denote labeled images from the source domain and unlabeled training images from the target domain, respectively, while ${\hat{x}}^{s\to t}$ and ${\hat{x}}^{t\to s}$ denote the translated samples with target-domain-like and source-domain-like styles. The target-domain-like samples generated by $G_{s\to t}$ inherit the source-domain annotations while preserving essentially unchanged geometric structures. Since BiDDC-Net performs pixel-aligned appearance/style transformation without changing object locations, scales, shapes, or image coordinates, the original bounding-box annotations remain spatially consistent with the generated target-domain-like images. Therefore, the source-domain bounding boxes can still be used as valid supervision for ${\tilde{x}}^{s\to t}$. Conversely, the source-domain-like samples generated $G_{t\to s}$ serve solely as unlabeled auxiliary samples during training and do not introduce any manual annotations from the target domain. For synthetic data with cross-weather correspondence, these pairwise relationships are not used as supervision signals during training. BiDDC-Net is employed only during training and is removed during inference, thereby avoiding additional testing overhead for the detector. Its overall structure is shown in Figure 2. It should be noted that, although training images from the source domain and target domain may correspond to the same scene in paired synthetic benchmarks, such correspondence is not used for supervision, pairwise loss construction, pseudo-label generation, or access to testing information. Therefore, the conclusions in this paper should be understood as results obtained under the current paired synthetic UDA setting, rather than as direct evidence for stricter unpaired real-world adaptation.

Structurally, BiDDC-Net adopts an encoder–decoder generator with skip connections and produces an output image with the same spatial resolution as the input. The input RGB image is progressively downsampled by multi-layer convolutions to produce 256-channel intermediate features, and a four-branch dynamic dilated convolution module is inserted at the end of the encoder. Subsequently, the spatial resolution is gradually restored through a two-stage deconvolution process, while skip connections are established with shallow and intermediate features to reduce the loss of local details. Importantly, BiDDC-Net does not apply cropping, affine transformation, perspective transformation, or other geometric warping operations. Therefore, it changes only the weather-related appearance/style while keeping the image geometry and bounding-box coordinates unchanged. In addition to the final output, the network also produces two intermediate reconstruction results at 1/4 and 1/2 resolutions to stabilize training. In the current implementation, the forward input of BiDDC-Net consists only of RGB images. If severe-weather samples are synthesized by a physical imaging model, the associated physical priors are reflected only in the construction of training samples, rather than being explicitly introduced as an additional network input branch.

To adapt to varying degrees of weather degradation, BiDDC-Net introduces degradation-aware dynamic dilated convolutions in the latter part of the encoder. Let the normalized input grayscale image be $x_{g}$. By Equation (4), its global brightness is defined as

(4) $$\mu \left(x_{g}\right)=\frac{1}{HW}\sum_{u=1}^{H}\sum_{v=1}^{W}x_{g}\left(u,v\right).$$

By Equation (5), its global contrast is defined as

(5) $$\sigma \left(x_{g}\right)=\sqrt{\frac{1}{HW}\sum_{u=1}^{H}\sum_{v=1}^{W}{\left(x_{g}\left(u,v\right)-\mu \left(x_{g}\right)\right)}^{2}}.$$

Based on this, we construct a heuristic degradation estimate r(x). It should be noted that r(x) is a lightweight proxy rather than a complete physical degradation model. It is mainly designed to capture fog-related, low-contrast, and darkening degradations, which are common in adverse-weather UAV and driving scenes. Brightness and contrast are adopted because they are simple, annotation-free, and directly related to the visibility loss caused by fog or illumination degradation. This design allows BiDDC-Net to estimate the degradation tendency with negligible computational cost and adjust the receptive fields accordingly. By Equation (6), r(x) is defined as

(6) $$r\left(x\right)=\mathrm{clip}\left(\left(0.5-\mu \left(x_{g}\right)\right)+\left(0.5-\sigma \left(x_{g}\right)\right),0,1\right).$$

where a larger value of r(x) indicates a darker image with lower contrast, i.e., a higher degree of degradation. Given four base dilation factors {2, 4, 8, 16}, the dynamic dilation factor for each branch is defined by Equation (7) as

(7) $$d_{i}\left(x\right)=\max\left(1,\left\lfloor d_{i}^{\left(0\right)}\left(1+r\left(x\right)\right)\right\rfloor \right).$$

The base set $d_{i}^{\left(0\right)}\in \left\{2,\;4,\;8,\;16\right\}$ is chosen based on sensitivity analysis as a compact logarithmic multi-scale configuration. Compared with smaller sets, e.g., {1, 2, 3, 4}, larger sets, e.g., {4, 8, 16, 32}, or linearly spaced sets, e.g., {2, 4, 6, 8}, it better balances small-object detail preservation and contextual aggregation. Smaller dilation factors help retain edges and fine textures, while larger ones provide wider context under foggy or low-contrast degradation. The branch outputs are concatenated and fused by a 1×1 convolution. Discrete dilation values are used instead of continuous ones because standard dilated convolution operates on integer sampling grids, and discrete choices reduce sensitivity to fluctuations in the degradation estimate. Thus, severe degradation enlarges the receptive field for context modeling, whereas mild degradation preserves smaller fields for detail retention. Nevertheless, since the degradation estimate mainly depends on brightness and contrast, this design is more suitable for fog or low-contrast scenes and may be limited under complex rain, snow, glare, or motion blur.

Overall, BiDDC-Net reduces the appearance differences between normal and adverse weather conditions through bidirectional sample expansion and degradation-aware receptive field adjustment, providing more stable cross-domain inputs for the subsequent AIFI-DA and SDA modules without altering the detector’s testing architecture.

### 3.3. AIFI-DA (Style-Aware Domain Adaptation Module)

**Figure 3:**
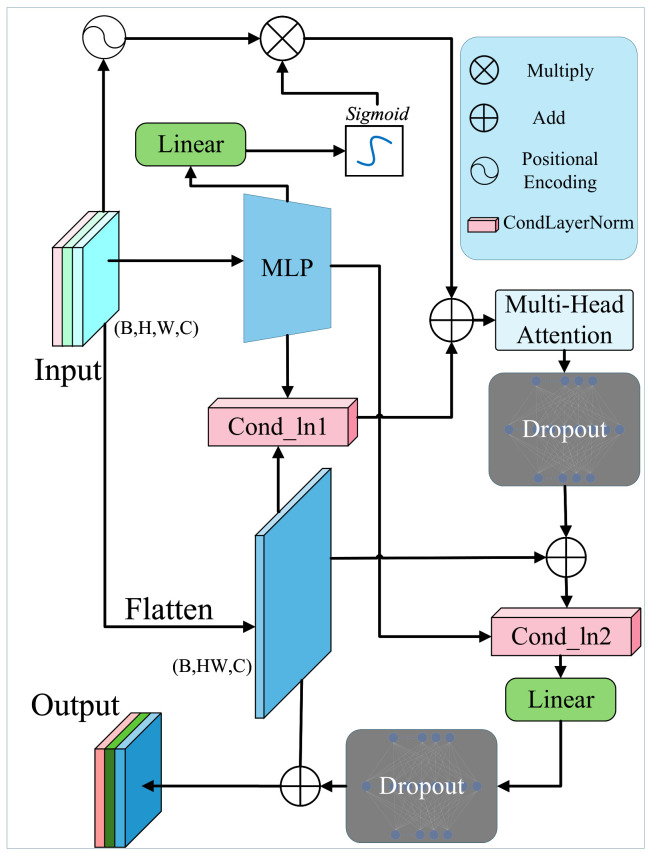
Schematic diagram of the AIFI-DA module. This module extracts statistical information from input features and processes it through an MLP to generate conditional vectors, which are used to dynamically adjust normalization parameters (Cond ln1/ln2) and position encoding scaling. By combining multi-head attention mechanisms with conditional self-attention, it achieves style-aware feature adaptation, enhancing cross-domain robustness while preserving the semantic meaning of the target.

While BiDDC-Net primarily mitigates appearance domain differences on the input side, blurring, low contrast, and texture weakening caused by adverse weather conditions still result in significant style shifts in mid-to-high-level features. To address this, we introduce a lightweight style-aware domain adaptation module, AIFI-DA, at the end of the backbone network to conditionally modulate high-level features and enhance the stability of cross-domain representations. Its specific structure is shown in Figure 3.

Let the input features be $F\in R^{C\times H\times W}$. AIFI-DA first extracts style descriptions based on channel statistics, concatenates the channel means and standard deviations, and inputs them into an MLP to obtain a style vector. By Equation (8), the style vector is defined as

(8) z=MLP([μ(F),σ(F)]).

This style vector is used to generate conditional normalization parameters for adaptive modulation of the input features. By Equation (9), the modulated feature is defined as

(9) $$\hat{F}=\gamma \left(z\right)⊙\mathrm{LN}\left(F\right)+\beta \left(z\right).$$

where LN(·) denotes layer normalization, and γ(z) and β(z) are style-related scaling and offset parameters, respectively. To enhance the stability of location modeling under different weather styles, this paper further utilizes *z* to scale the two-dimensional location codes. By Equation (10), the modulated location code is defined as

(10) $${\mathrm{PE}}^{\prime }\left(p\right)=\alpha \left(z\right)⊙\mathrm{PE}\left(p\right).$$

where PE(p) is the original location code at position *p*, and α(z) is the modulation coefficient generated by the style vector. Subsequently, the modulated features and location codes are jointly fed into a standard multi-head self-attention mechanism and a feedforward network to complete the cross-domain adaptation of high-level features. Since AIFI-DA operates only on low-resolution high-level feature maps, the additional computational overhead is minimal.

Overall, AIFI-DA mitigates style shifts in mid-to-high-level features caused by adverse weather through style-statistics-guided feature normalization and position encoding modulation, thereby providing consistent style representations for subsequent SDA.

### 3.4. SDA (Complementary Style Direction Alignment Regularizer)

Although AIFI-DA is capable of style-aware modulation of high-level features, using traditional alignment losses such as MSE or CORAL to directly constrain the numerical proximity between source-domain and target-domain features can impose overly strong constraints on the sparse representations of small objects. To provide a milder constraint, we introduce SDA as a training-time auxiliary regularizer. Instead of forcing source-domain and target-domain features to be numerically close, SDA only encourages the direction of cross-domain style variation to be consistent, thereby reducing potential over-constraint on small-object features. SDA is used only during training and does not introduce additional inference overhead.

For the selected *l* feature layer, let the source-domain and target-domain features be denoted as $F_{s}^{l}$ and $F_{t}^{l}$, respectively. Consistent with Section 3.3, we first construct a style vector based on channel statistics. By Equation (11), the source-domain style vector is defined as

(11) $$z_{s}^{l}=\left[\mu \left(F_{s}^{l}\right),\sigma \left(F_{s}^{l}\right)\right].$$

By Equation (12), the target-domain style vector is defined as

(12) $$z_{t}^{l}=\left[\mu \left(F_{t}^{l}\right),\sigma \left(F_{t}^{l}\right)\right].$$

Here, μ(·) and σ(·) represent the mean and standard deviation calculated per channel, respectively. The style vectors are then $L_{2}$ normalized to obtain the style direction. By Equation (13), the normalized source-domain style direction is defined as

(13) $${\bar{z}}_{s}^{l}=\frac{z_{s}^{l}}{{∥z_{s}^{l}∥}_{2}+\varepsilon }.$$

By Equation (14), the normalized target-domain style direction is defined as

(14) $${\bar{z}}_{t}^{l}=\frac{z_{t}^{l}}{{∥z_{t}^{l}∥}_{2}+\varepsilon }.$$

where $\varepsilon =10^{-6}$ is a constant used to prevent numerical instability. At a single scale, the SDA loss is defined as in Equation (15),

(15) $$L_{\mathrm{SDA}}=\lambda_{1}\cdot \frac{1}{K}\sum_{k=1}^{K}SDA^{\left(k\right)}.$$

where *K* is the number of feature scales involved in the calculation. SDA shares the core representation of “style vectors” with AIFI-DA. The style vectors generated in AIFI-DA can be directly used as input for SDA without the need for additional feature construction, thereby reducing computational redundancy and aligning the feature modulation objective with the loss optimization objective. Compared to methods such as CORAL and MSE that directly match statistical magnitudes, SDA focuses more on the consistency of style change directions between the source and target domains, rather than forcing them to be numerically identical. This helps alleviate over-constraints while preserving domain-specific characteristics.

In the total loss function, $L_{\mathrm{SDA}}$ is jointly optimized with the baseline detection loss, image-level domain adaptation loss, and pixel-level domain adaptation loss, with the overall form given by Equation (16) as

(16) $$L_{\mathrm{total}}=L_{\det}+\lambda_{2}\cdot L_{\mathrm{img}\text{-}\mathrm{da}}+\left(\lambda_{3}\cdot L_{\mathrm{pix}\text{-}\mathrm{src}}+\lambda_{4}\cdot L_{\mathrm{pix}\text{-}\mathrm{tgt}}\right)+L_{\mathrm{SDA}}.$$

where $L_{\det}$ is the base detection loss, $L_{\mathrm{img}\text{-}\mathrm{da}}$ is the image-level domain adaptation loss, $L_{\mathrm{pix}\text{-}\mathrm{src}}$ and $L_{\mathrm{pix}\text{-}\mathrm{tgt}}$ are the pixel-level source and target domain adaptation losses, respectively, and $\lambda_{1}$–$\lambda_{4}$ are the corresponding loss weights. Furthermore, SDA shares style vectors with AIFI-DA, reducing redundant computations while ensuring a high degree of alignment between the “feature modulation objective” and the “loss optimization objective.” SDA therefore acts as an auxiliary regularization term that complements AIFI-DA, rather than as an independent dominant adaptation module.

## 4. Experiments

### 4.1. Experimental Details

SABDR is implemented based on the YOLO detector. YOLO26 refers to the official Ultralytics YOLO26 detection model, and the YOLO26-based experiments in this study use the small variant initialized from yolo26s.pt. The detector keeps the original YOLO26s prediction structure with P3/8, P4/16, and P5/32 detection outputs. The YOLO26s configuration adopts the scale setting [0.50,0.50,1024], corresponding to depth and width scaling factors of 0.50 and 0.50 with a maximum channel number of 1024. Its backbone is composed of Conv and C3k2 blocks followed by SPPF and C2PSA, while the neck/head performs multi-scale feature fusion using upsampling, concatenation, Conv, and C3k2 blocks before the final Detect layer. We evaluate the proposed method on YOLOv5s and YOLO26; specifically, the Cityscapes→Foggy Cityscapes experiment is based on YOLOv5s, while the MOT-Fly→Weather MOT-Fly experiment is based on both YOLOv5s and YOLO26. Apart from the proposed adaptation module, each detector retains the backbone, neck, and detection head from its original implementation. In other words, SABDR does not alter the detector’s basic prediction framework but integrates the corresponding adaptation module into the respective YOLO detector.

In terms of specific implementation, SABDR inherits the basic cross-domain detection framework of R-YOLO and introduces three modifications. First, QTNet, used in the training phase of R-YOLO, is replaced with BiDDC-Net for bidirectional cross-domain sample augmentation on the input side; second, AIFI-DA is inserted between the backbone network output and the neck to perform style-aware modulation on high-level features; Finally, SDA is introduced during the training phase as an additional style-direction alignment loss. It should be noted that BiDDC-Net is enabled only during training and removed during testing; SDA is included only in the training loss calculation; and AIFI-DA is retained in both the training and testing phases. Therefore, BiDDC-Net and SDA do not introduce additional inference overhead during testing.

Regarding the training protocol, source-domain images are manually annotated, while target-domain images do not use any manual annotations during the training phase. All target-domain annotations are used solely for validation and testing and do not participate in UDA training. To ensure a fair comparison, Source-YOLO, R-YOLO, and SABDR under the same detector adopt consistent input dimensions, optimizer settings, training epochs, and testing strategies.

All input images are resized to 640 × 640, with a uniform batch size of 16, and trained for 300 epochs. The optimizer uses SGD with an initial learning rate of 0.01; other optimization hyperparameters follow the standard default settings in the respective detector implementations. Regarding model initialization, YOLOv5s uses the publicly released yolov5s.pt pre-trained weights, while YOLO26 is initialized using its corresponding yolo26s.pt. Except for the cross-domain images generated internally by the method, this paper does not employ additional data augmentation—i.e., Mosaic, MixUp, or other manual augmentation strategies—to minimize the interference of augmentation strategies on cross-domain adaptation performance. MOT-Fly is a one-class UAV detection dataset; therefore, the dataset configuration sets nc = 1. Although the base yolo26.yaml template contains nc = 80 for COCO-style pretraining, the class number is set to one during MOT-Fly training. All input images are resized to 640×640, and the YOLO26-based MOT-Fly experiments are trained for 300 epochs with a fixed batch size of 16.

In the loss function, the weights for SDA, image-level domain adaptation, pixel-level source domain alignment, and pixel-level target domain alignment are set to $\lambda_{1}=0.05$, $\lambda_{2}=0.1$, $\lambda_{3}=0.5$, and $\lambda_{4}=0.5$, respectively. Additionally, the decay coefficient for EMA is set to 0.999. During the inference stage, standard Non-Maximum Suppression (NMS) is employed, with confidence and IoU thresholds set to 0.25 and 0.6, respectively. All experiments were conducted using a fixed random seed of 42 on an Intel Xeon W-2245 CPU and NVIDIA RTX 3090 24GB GPU platform, running on Ubuntu 20.04 with PyTorch 1.11 and CUDA 11.3. Due to the high training cost of repeated cross-domain detection experiments, the reported MOT-Fly results are obtained from a single run under the fixed random seed 42, without multi-seed statistical validation. Therefore, these improvements should be interpreted as empirical observations rather than statistically significant differences.

For efficiency evaluation, inference is performed on the same Intel Xeon W-2245 CPU and NVIDIA RTX 3090 24GB GPU platform with an input size of 640 × 640 and an inference batch size of 1. Before timing, 50 warm-up iterations are conducted and excluded from the statistics. The inference latency is measured after CUDA synchronization and reported as milliseconds per image, while FPS is computed from the averaged inference time. Peak GPU memory is recorded using the maximum allocated CUDA memory during inference.

### 4.2. Datasets and Evaluation Metrics

Following the cross-domain detection setup described in the references, this paper evaluates SABDR on two cross-weather benchmark settings: Cityscapes→Foggy Cityscapes and MOT-Fly→Weather-Motfly. The former is used to validate cross-weather domain adaptability in urban foggy scenarios, while the latter is used to assess the cross-domain performance of drone small-object detection under the fog, rain, and snow settings included in Weather-Motfly. Unless otherwise specified, the source domain provides annotations, while the target domain, including Foggy Cityscapes and Weather-Motfly, does not use human annotations during the training phase; annotations in the target domain are used solely for evaluation.

**Cityscapes→Foggy Cityscapes:** Cityscapes is a commonly used urban scene benchmark in the autonomous driving field, comprising 2975 training images and 500 validation images. Following the standard setup in existing cross-domain detection studies, this paper selects 8 of these classes for evaluation. Foggy Cityscapes is generated from Cityscapes based on a standard atmospheric scattering model and provides three fog density settings (β=0.005,0.01,0.02). To maintain consistency with existing benchmarks and construct more pronounced cross-domain differences, this paper adopts the dense fog version with β=0.02 as the target domain and the original clear-sky Cityscapes as the source domain. The experiments strictly adhere to the official data partitioning; the clear-sky image and its foggy version for the same scene are always located in the same data subset.
**MOT-Fly→Weather MOT-Fly:** MOT-Fly is a dataset designed for small-object detection of UAVs, comprising 5496 clear-sky low-altitude images covering urban low-altitude, suburban low-altitude, and open-sky scenes, with the sole class being “uav”. The dataset is divided by scene into a training set of 3847 images, a validation set of 1099 images, and a test set of 550 images. Since the targets in the data are all small-scale UAVs, it is suitable as an evaluation benchmark for cross-domain detection of small targets under adverse weather conditions. To enhance the transparency of the experimental protocol, this paper constructs Weather MOT-Fly based on MOT-Fly as the weather-degraded target domain, including foggy, rainy, and snowy variants. Specifically, the foggy variant adopts the same atmospheric scattering generation method as Foggy Cityscapes and uses the same fog density parameter β=0.02 to generate dense fog images; therefore, the foggy MOT-Fly setting should be regarded as a controlled synthetic validation rather than a fully real-world fog benchmark. The rainy and snowy variants are newly constructed in this paper using the weather augmentation operators provided by Albumentations (https://github.com/albumentations-team/albumentations) (accessed on 3 June 2026), where the rainy variant is generated with RandomRain using rain_intensity = 0.55, and the snowy variant is generated with RandomSnow using snow_intensity = 0.80. All weather-degraded variants inherit the object categories, bounding-box annotations, and training/validation/test split protocol of the original MOT-Fly dataset, with only the image appearance modified to simulate weather-induced degradation. In other words, Weather MOT-Fly is not a separately designed complex real-weather dataset, but a MOT-Fly-based weather-degraded benchmark constructed for evaluating small-object cross-domain detection under the fog, rain, and snow settings considered in this paper.

**Figure 4:**
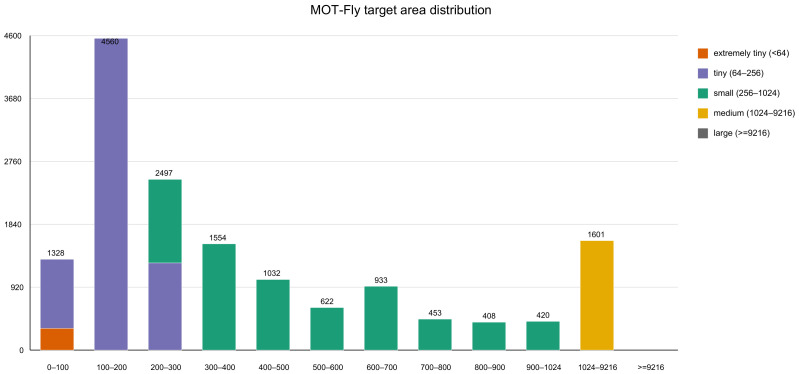
Target area distribution of the MOT-Fly dataset. All target-size statistics are computed after resizing the images and bounding-box annotations to the 640×640 input resolution used by the detector.

Following the commonly used COCO-style scale criterion, we define an object as a small target when its bounding-box area satisfies A=w×h<1024, corresponding approximately to an object smaller than 32×32 pixels^2^. All target-size statistics are computed after resizing the images and bounding boxes to the 640×640 input resolution used in our experiments, so that the scale distribution is consistent with the detector input. As shown in Figure 4, MOT-Fly contains 15,408 UAV instances in total, among which 13,807 instances have areas smaller than 1024 pixels^2^, accounting for 89.61% of all targets. In contrast, only 1601 instances fall into the medium-scale range of 1024–9216 pixels^2^, and no large targets larger than 9216 pixels^2^ are observed. The distribution is highly concentrated in the low-area intervals, especially the 100–300 pixels^2^ range, indicating that MOT-Fly is dominated by extremely small UAV targets and is therefore suitable for evaluating cross-domain small-object detection under the evaluated weather-degraded settings.

**Figure 5:**
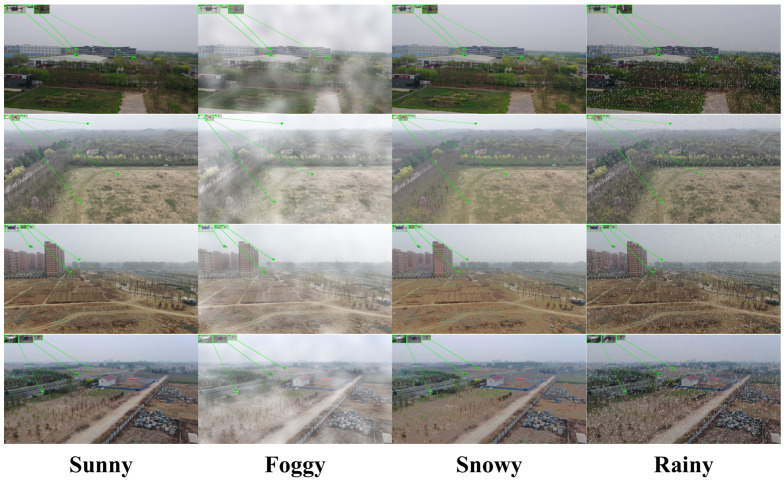
Example from the Weather-MotFly dataset. For clarity, the green boxes highlight the small target drone area in the figure, and the images show a 4x magnification of the corresponding region, representing samples under normal weather conditions and foggy weather conditions, respectively.

To avoid potential information leakage, this study follows the principle of “first partitioning into subsets, then generating weather-degraded images,” meaning that the original MOT-Fly dataset is first divided into training, validation, and test sets by scene, and then the foggy, rainy, and snowy images in Weather-Motfly are generated independently within each subset. Consequently, Weather-Motfly maintains a strict one-to-one correspondence with MOT-Fly, with training, validation, and test set sizes identical to those of the original dataset, comprising 3847, 1099, and 550 images, respectively, for each weather variant. Clear-sky images and their weather-degraded versions for the same scene are always located within the same data subset, ensuring that the training phase does not indirectly expose the content of target-domain validation or test images through clear-sky images. Furthermore, Weather-Motfly maintains consistency with MOT-Fly in terms of object categories, bounding box locations, and annotation information; only the weather conditions have changed. Target-domain annotations are used solely for validation and testing and do not participate in unsupervised domain adaptation training. An example of the Weather-MOTfly dataset is shown in Figure 5.

Given the differing task attributes of the two benchmarks, this paper adopts evaluation metrics that align with their respective roles. The Cityscapes-to-Foggy Cityscapes setting is used as a reference benchmark for comparison with general cross-domain object detection methods, and the corresponding results are reported in Table 1. Therefore, we follow the commonly used evaluation protocol of this benchmark and report the overall ${\mathrm{mAP}}_{50}$, rather than scale-wise ${\mathrm{AP}}_{s}$/${\mathrm{AP}}_{m}$/${\mathrm{AP}}_{l}$ scores, to evaluate the method’s general cross-weather adaptability. In contrast, MOT-Fly is used as the main benchmark for validating small-object cross-domain detection, with the results reported in Table 2. According to the COCO-style small-object criterion, i.e., A=w×h<1024 pixels^2^, MOT-Fly contains 15,408 annotated instances, among which 13,807 are small objects, accounting for 89.61% of all instances. This small-object-dominated distribution makes MOT-Fly the primary evidence for evaluating the proposed method under small-object domain shifts. Accordingly, for MOT-Fly→Weather MOT-Fly, this paper reports ${\mathrm{mAP}}_{50}$ and ${\mathrm{mAP}}_{50\text{-}95}$, as well as Params and GFLOPs, to comprehensively evaluate both small-object detection performance and model computational cost.

### 4.3. Comparison with State-of-the-Art Approaches

#### 4.3.1. Cityscapes and Foggy Cityscapes

To validate the effectiveness of SABDR on a public, cross-weather, unsupervised domain adaptation benchmark, this paper first conducts comparative experiments on Cityscapes→Foggy Cityscapes. Specifically, Cityscapes-train serves as the labeled source domain, and Foggy Cityscapes (β=0.02) serves as the unlabeled target domain; all methods are evaluated on Foggy Cityscapes-val. Following the common protocol in existing work, this paper reports $AP_{50}$ across 8 categories as well as the overall ${\mathrm{mAP}}_{50}$, with ${\mathrm{mAP}}_{50}$ serving as the primary evaluation metric in this section. Therefore, Table 1 separates strictly controlled comparisons from literature-reference comparisons. Source-YOLOv5, R-YOLO, and SABDR use the same detector, training protocol, and evaluation setting, whereas the other methods are cited from published papers and may differ in backbone, detector architecture, training schedule, and computational budget.

As shown in Table 1, Source-YOLOv5, which is trained using only source-domain annotations and tested directly on the target domain, achieves an ${\mathrm{mAP}}_{50}$ of 16.4, indicating that the visual degradation and domain shift caused by the transition from clear weather to dense fog significantly degrade detection performance. After introducing a cross-domain adaptation mechanism, R-YOLOv5 improves the ${\mathrm{mAP}}_{50}$ to 45.2, showing that the baseline UDA framework can effectively mitigate the aforementioned distribution differences. Building on this, SABDR further achieves an ${\mathrm{mAP}}_{50}$ of 47.7, representing improvements of 31.3 and 2.5 percentage points over Source-YOLOv5 and R-YOLOv5, respectively. It should be emphasized that the strictly fair comparison in this experiment is among Source-YOLOv5, R-YOLOv5, and SABDR, since they are implemented with the same YOLOv5s-based detector, training protocol, and evaluation setting. The comparisons with other published methods in Table 1 are provided as reference comparisons, because these methods may differ in backbone, detector type, model scale, training strategy, and computational cost. Therefore, SABDR’s result should be interpreted as competitive under the reported benchmark setting rather than as an absolute claim of superiority over all existing methods. In recent years, larger Transformer-based or vision foundation models, such as DINO-family detectors, Grounding DINO, and detection frameworks based on DINOv2/3-style representations, have shown strong potential in general detection tasks. However, these methods usually rely on much larger model capacities and computational budgets, and are not under the same lightweight resource constraints as the YOLOv5s-based SABDR; thus, they are not included as direct comparison targets under strictly equivalent protocols.

In terms of category-wise results, SABDR outperforms R-YOLOv5 in 7 out of 8 categories, with more pronounced gains in the truck and train categories, while a slight decrease in $AP_{50}$ is observed for the bus category. These results suggest that the proposed BiDDC-Net, AIFI-DA, and SDA can generally enhance cross-domain detection performance from clear to foggy conditions; however, the degree of improvement varies across categories and remains influenced by factors such as target size distribution, scene context, and the severity of weather degradation. Overall, SABDR demonstrates relatively consistent cross-weather adaptation gains on this public benchmark.

It should be emphasized that Cityscapes→Foggy Cityscapes is a standard public benchmark containing objects at multiple scales. Therefore, experiments on this dataset are intended to validate the general cross-weather domain adaptation capability of the proposed method, rather than to exclusively demonstrate its small-object detection performance. More quantitative evidence regarding small-object detection under adverse weather conditions will be provided in the next subsection on the MOT-Fly→Weather MOT-Fly all-small-object benchmark.

**Figure 6:**
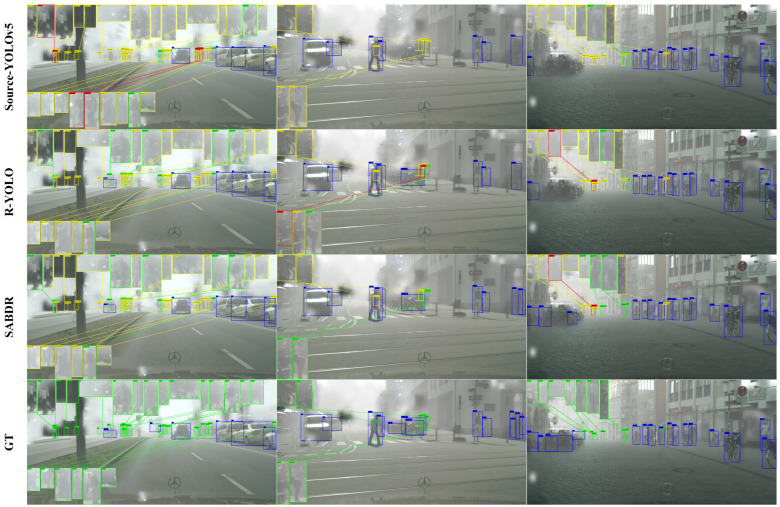
Visual comparison of object detection results from different algorithms in the cross-domain scenario from Cityscapes to Foggy Cityscapes. The blue boxes indicate ground truth for targets of normal size; the yellow, green, and red boxes represent detection results for small targets, where green indicates a true positive (TP), yellow indicates a false negative (FN), and red indicates a false positive (FP).

To visually demonstrate the detection differences among different methods on the target domain, Figure 6 presents several visualization results on the Foggy Cityscapes validation set. Compared with Source-YOLOv5, both R-YOLOv5 and SABDR significantly reduce false negatives in low-contrast scenes. Furthermore, SABDR generally provides more stable predictions for distant objects heavily affected by fog and reduces false positives in some complex background regions. Nevertheless, a small number of challenging cases can still be observed in the figures, indicating that cross-weather detection on this public benchmark remains highly challenging. The visual results in Figure 6 serve primarily as a qualitative supplement and should be interpreted together with the quantitative results reported in Table 1.

#### 4.3.2. MOT-Fly and Weather MOT-Fly

To further validate the small-object detection performance of SABDR under the evaluated fog/rain/snow settings, this paper conducted comparative experiments between MOT-Fly and Weather-Motfly. Specifically, the training subset of MOT-Fly was used as the labeled source domain, while the corresponding training subsets of Weather-Motfly served as unlabeled target domains, and results were reported on the target-domain evaluation subsets. To avoid potential information leakage, Weather-Motfly follows a “split-first, degrade-later” protocol: the original MOT-Fly data are first divided into training, validation, and test subsets, and the corresponding weather-degraded images are then generated independently within each subset. As a result, each clear-sky image and its foggy, rainy, or snowy counterparts remain in the same data subset. During training, only source-domain annotations and unlabeled target-domain images are used, with no manual annotations from the target domain. Since Weather-Motfly is constructed through controlled weather-degradation protocols, the experimental conclusions mainly reflect the effectiveness of the proposed method under the evaluated fog, rain, and snow settings, rather than its generalization to all real-world adverse-weather conditions.

Unlike Cityscapes→Foggy Cityscapes, where objects appear at multiple scales, all targets in MOT-Fly→Weather MOT-Fly are small-scale drones. Therefore, the overall ${\mathrm{mAP}}_{50}$ and ${\mathrm{mAP}}_{50\text{-}95}$ on this benchmark directly reflect the method’s performance in detecting small objects under foggy conditions. To verify the consistency of the proposed method across different detectors, experiments were conducted using both YOLOv5s and YOLO26. For each detector, Source-YOLO, R-YOLO, and SABDR were trained and evaluated under identical input resolutions, training epochs, optimizer settings, and evaluation protocols. In addition, Table 2 reports Params and GFLOPs as supplementary indicators of computational overhead.

**Table 2:** Single-run empirical results (%) for adaptation from MOT-Fly to Weather MOT-Fly under a fixed random seed. Gray denotes foggy images, blue denotes rainy images, and off-white denotes snowy images.

Method	Params	GFLOPs	${\mathrm{mAP}}_{50}$	${\mathrm{mAP}}_{50\text{-}95}$	${\mathrm{mAP}}_{50}$	${\mathrm{mAP}}_{50\text{-}95}$	${\mathrm{mAP}}_{50}$	${\mathrm{mAP}}_{50\text{-}95}$
Source-YOLOv5	7,012,822	15.8	86.2	53.1	44.9	22.3	84.6	46.5
R-YOLO	7,566,936	18.3	94.8	62.9	65.3	34.2	88.9	54.2
SABDR	10,521,816	19.8	96.0	64.5	66.7	41.0	95.0	63.6
Source-YOLO26	9,465,567	20.5	88.8	61.1	51.4	29.1	93.8	62.5
R-YOLO	10,583,985	26.6	96.4	68.1	75.1	45.2	94.5	64.4
SABDR	11,785,969	28.2	96.8	68.3	77.1	47.5	95.6	64.4

As shown in Table 2, SABDR consistently improves detection performance on Weather MOT-Fly. On YOLOv5s, Source-YOLOv5 obtains ${\mathrm{mAP}}_{50}$ values of 86.2, 44.9, and 84.6 under foggy, rainy, and snowy conditions, respectively. R-YOLO increases these values to 94.8, 65.3, and 88.9, while SABDR further achieves 96.0, 66.7, and 95.0, corresponding to gains of 9.8/21.8/10.4 and 1.2/1.4/6.1 percentage points over Source-YOLOv5 and R-YOLO. A similar trend is observed on YOLO26, where SABDR reaches 96.8, 77.1, and 95.6 ${\mathrm{mAP}}_{50}$, improving over Source-YOLO26 and R-YOLO by 8.0/25.7/1.8 and 0.4/2.0/1.1 percentage points, respectively. Given the already high performance of R-YOLO on Foggy MOT-Fly, the additional gain achieved by SABDR provides single-run empirical evidence of effectiveness in a saturated performance regime, but it does not, by itself, establish statistical significance.

Table 3 reports the inference-time efficiency of R-YOLO and SABDR under FP16 precision with a 640×640 input size and a batch size of 1. FPS, latency, and peak GPU memory are measured after warm-up and CUDA synchronization. The results further reflect the impact of SABDR on runtime overhead. Since BiDDC-Net is enabled only during training and SDA participates only in the training loss calculation, these two modules are removed from the inference graph and do not introduce additional testing-time overhead. The additional inference cost mainly comes from AIFI-DA, which is retained during inference for feature modulation. Specifically, on YOLOv5s, R-YOLO achieves 46.905 FPS with 21.320 ms/img latency and 183.2 MB peak GPU memory, while SABDR achieves 18.776 FPS with 53.261 ms/img latency and 214.1 MB peak GPU memory. On YOLO26s, R-YOLO achieves 26.132 FPS with 38.268 ms/img latency and 200.5 MB peak GPU memory, while SABDR achieves 24.561 FPS with 40.715 ms/img latency and 217.6 MB peak GPU memory. These results indicate that the inference overhead of SABDR is mainly introduced by AIFI-DA, whereas BiDDC-Net and SDA only affect the training stage.

Overall, under the same single-seed experimental setting, SABDR achieves slightly higher ${\mathrm{mAP}}_{50}$ and ${\mathrm{mAP}}_{50\text{-}95}$ than R-YOLO on both YOLOv5s and YOLO26 detectors. These results suggest that the proposed BiDDC-Net, AIFI-DA, and SDA can provide additional improvements for small-object detection in foggy conditions, but the margin over the strong R-YOLO baseline remains limited. Therefore, Foggy MOT-Fly serves as useful quantitative evidence for evaluating the proposed small-object adaptation design, while stronger conclusions will require multi-seed validation and broader real-world adverse-weather testing.

**Figure 7:**
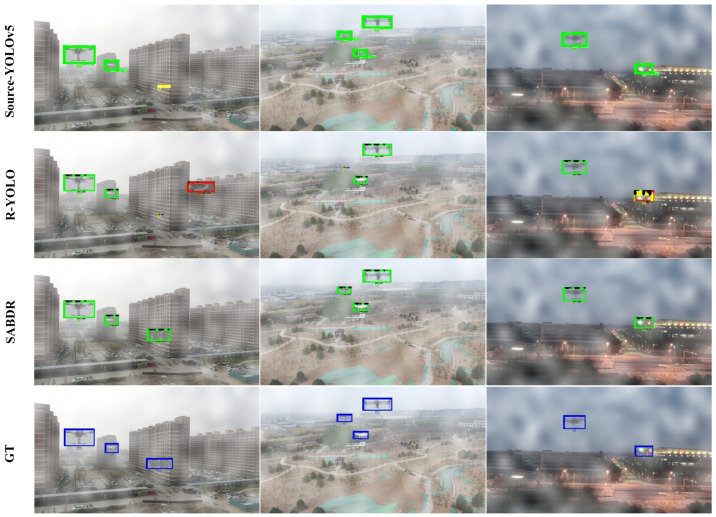
Comparison of cross-domain detection performance among different algorithms on the Foggy MOTFly dataset. From top to bottom: Source-YOLOv5 (source-domain training), R-YOLO, SABDR, and ground truth (GT). Blue boxes denote normal-sized ground truth; Green boxes indicate true positives (TP), red boxes indicate false positives (FP), and yellow boxes indicate false negatives (FN). To facilitate observation of small object details, all images are annotated with the regions of small objects, corresponding to 8x magnified views.

To further illustrate the differences in practical detection performance among different methods, Figure 7 presents several visualization examples from Foggy MOT-Fly. Compared with Source-YOLO, both R-YOLO and SABDR reduce false negatives under dense-fog conditions. Moreover, SABDR generally produces more stable predictions in challenging small-drone scenarios characterized by long distances, low contrast, and strong background interference, while also reducing false positives in some difficult cases. It should be noted that the visualizations in Figure 7 serve primarily as qualitative supplements, whereas the main evaluation of small-object detection performance in this paper is based on the quantitative results reported in Table 2.

### 4.4. Ablation Studies

#### 4.4.1. Ablation Study on the Hyperparameter

To determine the optimal weights of the loss terms, hyperparameter ablation experiments were performed on the Cityscapes→Foggy Cityscapes benchmark with respect to $\lambda_{1}$ (SDA loss weight), $\lambda_{2}$ (image-level domain adaptation weight), $\lambda_{3}$ (pixel-level source-domain alignment weight), and $\lambda_{4}$ (pixel-level target-domain alignment weight). The experimental results are presented in Table 3. Except for the loss weights being investigated, all remaining training settings were kept identical to those described in Section 4.1. According to a comprehensive assessment of ${\mathrm{mAP}}_{50}$, training stability, and convergence performance, SABDR attains the best overall detection results when $\lambda_{1}=0.05$, $\lambda_{2}=0.1$, and $\lambda_{3}=\lambda_{4}=0.5$.

#### 4.4.2. Ablation Study on the Modules

To evaluate the relative contributions of BiDDC-Net, AIFI-DA, and SDA, we performed module ablation experiments based on the R-YOLO baseline. As this work considers both a public cross-weather benchmark and an all-small-object UAV benchmark, Table 4 presents the ablation results on Cityscapes→Foggy Cityscapes (*c2f*) and MOT-Fly→Foggy MOT-Fly (*m2f*). The *c2f* benchmark is adopted to measure general cross-weather domain adaptation capability, whereas the *m2f* benchmark is introduced to verify whether the performance gains of each module remain consistent under adverse-weather small-object detection scenarios. Following the settings in Section 4.1, all experiments were conducted under the same training protocol and with a fixed random seed to ensure fair comparisons across different module combinations. We report *P*, *R*, ${\mathrm{mAP}}_{50}$, and ${\mathrm{mAP}}_{50\text{-}95}$ on both benchmarks, while Params and GFLOPs are provided as supplementary indicators of model efficiency.

These ablation results confirm that SDA should be interpreted as a complementary regularizer, not as a main standalone contribution. As shown in Table 5, the baseline R-YOLO achieves 45.2 on *c2f* and 94.8 on *m2f* in terms of ${\mathrm{mAP}}_{50}$. After introducing BiDDC-Net alone, the performance increases to 45.4 on *c2f* and 95.2 on *m2f*. This indicates that input-side bidirectional cross-domain sample augmentation and degradation-aware receptive field adjustment can alleviate the appearance gap between clear and foggy conditions on both benchmarks. Although the standalone gain is limited, BiDDC-Net provides a stable input-side enhancement and improves sample robustness before feature extraction. More importantly, when combined with AIFI-DA and SDA, it serves as an input-side stabilizer that helps the feature-level and loss-level adaptation modules work more effectively, thereby contributing to the overall performance of SABDR.

When AIFI-DA is introduced alone, the performance rises to 46.8 on *c2f* and 95.4 on *m2f*, representing the strongest gain among all single-module settings on both benchmarks. This result indicates that style-aware feature modulation between the backbone and the neck is the most effective standalone component for handling weather-induced style shifts. By contrast, adding SDA alone yields 45.3 on *c2f* and 95.0 on *m2f*, which is only slightly better than the baseline. This suggests that style-direction alignment is helpful, but its effect is more limited when used independently and becomes more effective when combined with stronger input- or feature-level adaptation.

Among the two-module combinations, BiDDC-Net + AIFI-DA achieves 47.3 on *c2f* and 95.8 on *m2f*, which is the best result among all two-module settings. This indicates strong complementarity between input-side sample augmentation and feature-side style modulation. BiDDC-Net + SDA reaches 45.7 on *c2f* and 95.3 on *m2f*, while AIFI-DA + SDA reaches 46.9 on *c2f* and 95.5 on *m2f*. These results further show that SDA contributes more effectively when combined with BiDDC-Net or AIFI-DA rather than acting as a dominant standalone module. Its main value comes from its synergy with input-side appearance augmentation and feature-side style modulation, where it provides an auxiliary directional constraint to improve the overall consistency of cross-domain adaptation. Notably, the relative ranking of the single-module and two-module settings is highly consistent across *c2f* and *m2f*, suggesting that the roles of the three modules are stable across different cross-weather detection scenarios.

When all three modules are enabled, SABDR achieves the best overall performance, reaching 47.7 on *c2f* and 96.0 on *m2f*. This corresponds to improvements of 2.5 and 1.2 percentage points over the baseline R-YOLO, respectively, as well as further gains of 0.4 and 0.2 points over the best two-module configuration. Although the absolute gain on *m2f* is smaller than that on *c2f*, this is mainly because the baseline performance on *m2f* is already very high, leaving less room for further improvement. Therefore, the full model’s consistent gains on both benchmarks provide stronger evidence that BiDDC-Net, AIFI-DA, and SDA form a complementary collaboration mechanism that is effective not only for general cross-weather adaptation but also for all-small-object detection under foggy conditions. Based on the current ablation results, AIFI-DA provides the primary direct gain, while BiDDC-Net and SDA mainly enhance overall performance through collaboration and refinement.

The Params and GFLOPs results in Table 5 also reflect the impact of each module on inference overhead. Since BiDDC-Net is enabled only during training and SDA participates only in the training loss calculation, adding only these two modules keeps the inference-time parameter count and computation essentially unchanged from the baseline. The additional inference overhead mainly comes from AIFI-DA. Specifically, the full SABDR contains 10.52 M parameters and 19.8 GFLOPs, compared with 7.57 M parameters and 18.3 GFLOPs for the baseline R-YOLO, indicating that the performance gains on both *c2f* and *m2f* are achieved at a relatively manageable inference cost.

**Figure 8:**
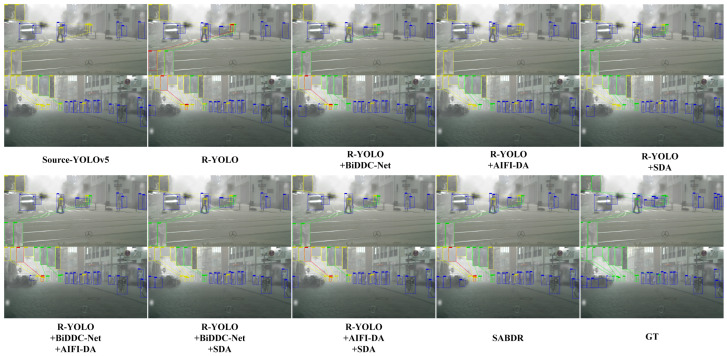
Visual comparison of object detection results from ablation experiments on Cityscapes→Foggy Cityscapes. Blue boxes denote normal-sized ground truth; for small objects, green, yellow, and red indicate TP, FN, and FP, respectively.

To further demonstrate the qualitative differences resulting from different module combinations, Figure 8 presents visualization results on Cityscapes→Foggy Cityscapes. Compared with the baseline R-YOLO, the configuration incorporating AIFI-DA typically yields more stable predictions for low-contrast and distant targets; BiDDC-Net helps recover some missed detections under foggy degradation and produces more stable localization boundaries for low-contrast targets; and the full SABDR configuration demonstrates a better overall balance between false negatives and false positives. Compared with the baseline R-YOLO, the configuration incorporating AIFI-DA typically yields more stable predictions for low-contrast and distant targets; BiDDC-Net helps reduce some false negatives caused by foggy degradation; and the full SABDR configuration demonstrates a better overall balance between false negatives and false positives. Combined with the consistent quantitative gains on *m2f* shown in Table 5, these observations support the effectiveness of the proposed modules across both general cross-weather scenes and all-small-object foggy detection settings. However, a small number of challenging instances can still be observed in the figure, indicating that domain-adaptive detection tasks under adverse weather remain highly challenging. The visualizations in Figure 8 serve primarily as a qualitative supplement and should be interpreted in conjunction with the quantitative results in Table 5.

## 5. Visualization and Limitations

### 5.1. Visualization

Combining Figure 6 and Figure 7, the visualization results show that SABDR produces more stable detection outputs in clear-to-adverse-weather cross-domain scenarios. On Cityscapes→Foggy Cityscapes, Source-YOLOv5 tends to miss objects in low-contrast or long-range regions, while R-YOLO alleviates part of the domain shift but still shows occasional false positives or localization deviations in complex backgrounds. In comparison, SABDR provides more consistent predictions, especially for fog-affected distant targets and objects in low-contrast regions.

A similar trend can be observed on MOT-Fly→Weather MOT-Fly. Since UAV targets are small and visually weak under adverse-weather conditions such as fog, rain, and snow, Source-YOLO and R-YOLO are more likely to produce missed detections in challenging cases. SABDR maintains more continuous detection outputs and more stable localization in the visual examples, indicating its improved robustness for small-object detection under cross-weather domain shifts.

**Figure 9:**
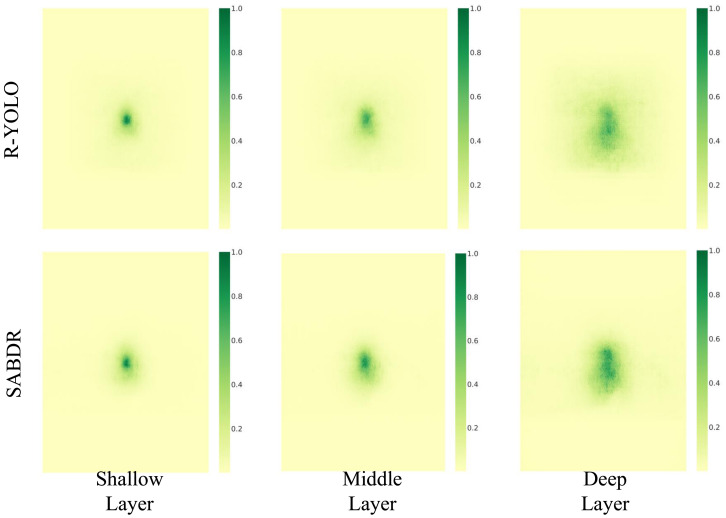
Visualization of the receptive fields of R-YOLO and SABDR at the feature level (heatmaps).

To better explain Figure 9, we clarify that the ERF heat maps are generated using a gradient-based method on the selected feature-pyramid layer. Each input image is resized to 640×640 and normalized to [0,1]. The positive activation at the central spatial location of the selected feature layer is back-propagated to the input image, and the positive input gradients are summed over RGB channels to form a contribution map. The maps from 50 images are then averaged, log-transformed, and normalized to [0,1]. Brighter regions indicate input pixels that contribute more strongly to the central feature response, showing the model’s effective receptive field and contextual aggregation ability.

Figure 9 further presents the heatmap responses of R-YOLO and SABDR at different feature levels. The visualization indicates that SABDR tends to generate more concentrated responses around target regions. At shallow layers, the responses are more closely related to local edges and texture details; at middle layers, the target-background transition areas become clearer; and at deeper layers, the contextual response around the target appears more stable. These heatmap results visually suggest that SABDR can enhance target-related feature responses under adverse-weather cross-domain settings.

### 5.2. Limitations

**Figure 10:**
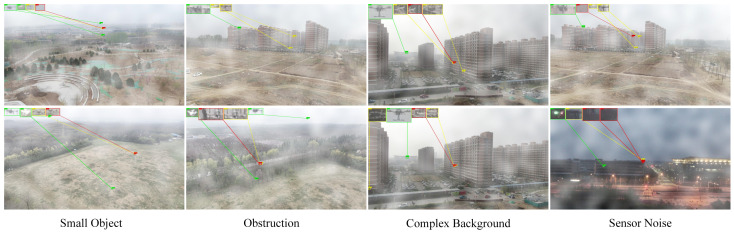
Failure cases of SABDR on Foggy MOT-Fly. The examples show typical challenging cases, including extremely small objects, obstruction, complex backgrounds, and sensor noise, where SABDR may still produce missed detections or false positives under foggy conditions.

Although SABDR achieves consistent improvements under the evaluated fog/rain/snow settings, it still has several limitations in challenging scenarios. As shown in Figure 10, typical failure cases occur when targets are extremely small or point-like, partially or heavily obstructed, affected by strong sensor noise, or embedded in complex backgrounds. In these cases, the discriminative cues of small objects become weak or ambiguous, and the detector may produce missed detections or false positives. In addition, the current experiments are mainly conducted on synthetic fog and limited UAV weather settings, so the robustness to real, mixed, and non-uniform adverse weather still requires further validation.

Qualitative results show that SABDR may still fail under heavy obstruction, strong sensor noise, extremely dense fog, complex backgrounds, and extremely small or point-like targets, as shown in Figure 10. These failures are mainly caused by weakened or ambiguous small-object cues, including reduced visible regions, degraded texture and boundary information, background distractors, and limited spatial features.

One limitation is that we do not report ${\mathrm{AP}}_{s}$/${\mathrm{AP}}_{m}$/${\mathrm{AP}}_{l}$ for the Cityscapes→Foggy Cityscapes results in Table 1. This setting is included mainly to provide a reference comparison with general cross-domain detection algorithms, rather than to serve as a dedicated scale-wise evaluation benchmark for small objects. The small-object evidence in this work is instead mainly supported by MOT-Fly, whose results are shown in Table 2 and whose annotations are dominated by small instances: 13,807 out of 15,408 instances, or 89.61%, satisfy A=w×h<1024 pixels^2^. Future work may further extend the evaluation protocol to include scale-wise AP on additional benchmarks when the dataset and comparison methods are specifically designed for small-object scale analysis.

## 6. Conclusions

This paper proposes the SABDR framework for small-object cross-domain detection under the evaluated foggy Cityscapes and Weather MOT-Fly settings. SABDR consists of three collaborative components—BiDDC-Net, AIFI-DA, and SDA—which address domain shift and small-object feature degradation from the input/sample layer, feature layer, and training objective layer, respectively. By combining input-side sample augmentation, feature-side style modulation, and loss-side style-direction constraints, SABDR improves cross-weather adaptability while preserving sparse and fragile small-object discriminative cues. Among the two main modules and one complementary regularizer, AIFI-DA provides the most direct standalone contribution by modulating style-related feature statistics, while SDA mainly serves as a training-time auxiliary directional constraint that works more effectively in collaboration with BiDDC-Net and AIFI-DA. Therefore, the main contribution of SABDR lies in this targeted three-level collaborative design rather than in any single dominant module.

Experimental results demonstrate that SABDR achieves improvements on both Cityscapes→Foggy Cityscapes and MOT-Fly→Weather MOT-Fly. Compared with the corresponding baselines, SABDR obtains higher ${\mathrm{mAP}}_{50}$ under the evaluated foggy, rainy, and snowy conditions, indicating its ability to mitigate weather-induced domain drift. On MOT-Fly, where the baseline performance is already high, SABDR brings single-run modest empirical gains under the evaluated single-seed setting, and these results should therefore be interpreted conservatively. Ablation studies further show that AIFI-DA provides the most direct standalone gain, while BiDDC-Net and SDA contribute more effectively when combined with other modules. The similar behavior of different module combinations across the two benchmarks also supports the stability of the proposed collaborative design.

Despite these encouraging results, several limitations remain. Cityscapes→Foggy Cityscapes is largely based on synthetic fog, and the MOT-Fly weather settings still have limited diversity in weather intensity, scene layout, flight altitude, and sensor conditions. Therefore, the generalization of SABDR to real, mixed, and non-uniform adverse weather requires further validation. In addition, qualitative results show that SABDR may still fail in challenging cases involving heavy occlusion, strong sensor noise, extremely dense fog, complex backgrounds, and extremely small or point-like targets, where discriminative cues become weak or ambiguous. Moreover, AIFI-DA introduces additional inference latency and GPU memory consumption, SDA has limited standalone contribution, and BiDDC-Net may be insufficient for modeling more complex degradations beyond brightness and contrast variations.

Based on these limitations, the conclusions of this paper should be interpreted within the evaluated fog/rain/snow cross-weather settings. Future work will be conducted in the following areas:

1. We will collect real-world UAV data under foggy, rainy, and snowy conditions, and further evaluate SABDR in mixed and non-uniform adverse-weather scenarios to verify its generalization capability in more realistic environments.
2. We will conduct experiments across multiple random seeds and introduce more complete statistical analyses and efficiency evaluations to further improve the reliability and comprehensiveness of the experimental report.
3. We will explore temporal modeling, motion constraints, few-shot or test-time adaptation mechanisms, and lightweight degradation-aware modules to improve robustness while reducing computational cost and inference latency.

## Figures and Tables

**Table 1:** Results (%) from Cityscapes to Foggy Cityscapes. Only Source-YOLOv5, R-YOLO, and SABDR are strictly controlled comparisons under the same YOLOv5s-based setting; other published methods are reference comparisons under different settings.

Method	Type	Cityscapes→Foggy Cityscapes								
		Person	Rider	Car	Truck	Bus	Train	Mcycle	Bicycle	${\mathrm{mAP}}_{50}$
DAF [23]	CNN	48.2	48.8	61.5	22.6	43.1	20.2	30.3	42.1	39.6
SWF [24]	CNN	49.0	49.0	61.4	23.9	43.1	22.9	31.0	45.2	40.7
GPA [20]	CNN	49.5	46.7	58.6	26.4	42.2	32.3	29.1	41.8	40.8
CRDA [33]	CNN	49.8	48.4	61.9	22.3	40.7	30.0	29.9	45.4	41.1
SFA [34]	Transformer	46.5	48.6	62.6	25.1	46.2	29.4	28.3	44.0	41.3
MTTrans [35]	Transformer	47.7	49.9	65.2	25.8	45.9	33.8	32.6	46.5	43.4
AQT [36]	Transformer	49.3	52.3	64.4	27.7	53.7	46.5	36.0	46.4	47.1
DA-DETR [37]	Transformer	49.9	50.0	63.1	24.0	45.8	37.5	31.6	46.3	43.5
MTOR [38]	Transformer	30.6	41.4	44.0	21.9	38.6	40.6	28.3	35.6	35.1
MSA [39]	Transformer	32.3	44.0	46.8	20.8	43.3	45.8	29.7	33.4	37.0
CTBL [40]	Transformer	41.7	33.2	65.0	32.5	43.2	35.8	31.2	30.9	39.2
Source-YOLOv5	CNN	27.3	25.3	33.2	4.3	10.7	1.5	9.2	20.1	16.4
R-YOLO	CNN	45.3	49.4	66.6	32.1	52.9	42.7	34.5	39.2	45.2
SABDR	CNN	46.4	50.1	67.5	36.3	50.8	54.0	35.5	40.5	47.7

**Table 3:** Inference-time efficiency comparison of R-YOLO and SABDR.

Method	Detector	Precision	FPS	Latency (ms/Img)	Peak GPU Memory (MB)
R-YOLO	YOLOv5s	FP16	46.905	21.320	183.2
SABDR	YOLOv5s	FP16	18.776	53.261	214.1
R-YOLO	YOLO26s	FP16	26.132	38.268	200.5
SABDR	YOLO26s	FP16	24.561	40.715	217.6

**Table 4:** SABDR Hyperparameter Ablation Results (%).

$\lambda_{2},\lambda_{3},\lambda_{4}$	$\lambda_{1}$	${\mathrm{mAP}}_{50}$	$\lambda_{1},\lambda_{3},\lambda_{4}$	$\lambda_{2}$	${\mathrm{mAP}}_{50}$	$\lambda_{1},\lambda_{2}$	$\lambda_{3}=\lambda_{4}$	${\mathrm{mAP}}_{50}$
$\lambda_{2}=0.1$ $\lambda_{3}=\lambda_{4}=0.5$	0.02	46.8	$\lambda_{1}=0.05$ $\lambda_{3}=\lambda_{4}=0.5$	0.05	46.0	$\lambda_{1}=0.05$ $\lambda_{2}=0.1$	0.25	46.1
	0.03	47.3		0.07	47.2			
	0.04	46.2		0.08	46.5		0.5	47.7
	0.05	47.7		0.09	46.1			
	0.06	46.4		0.1	47.7			
	0.07	46.3		0.15	45.6		0.75	47.3
	0.08	45.5		0.2	47.2			

**Table 5:** Ablation results (%) of SABDR modules.

R-YOLO	BiDDC-Net	AIFI-DA	SDA	Params	GFLOPs	${\mathrm{mAP}}_{50}$ (c2f)	${\mathrm{mAP}}_{50}$ (m2f)
🗸				7,566,936	18.3	45.2	94.8
🗸	🗸			7,566,936	18.3	45.4	95.2
🗸		🗸		10,521,816	19.8	46.8	95.4
🗸			🗸	7,566,936	18.3	45.3	95.0
🗸	🗸	🗸		10,521,816	19.8	47.3	95.8
🗸	🗸		🗸	7,566,936	18.3	45.7	95.3
🗸		🗸	🗸	10,521,816	19.8	46.9	95.5
🗸	🗸	🗸	🗸	10,521,816	19.8	**47.7**	**96.0**

Note: 🗸 indicates that the corresponding module is used. Bold values indicate the best performance.

## Data Availability

Publicly available datasets were analyzed in this study, including Cityscapes and Foggy Cityscapes. Information regarding other datasets and experimental materials is available from the corresponding author upon reasonable request.
